# Fractional Flow Reserve Measurement Using Dynamic CT Perfusion Imaging in Patients with Heavy Coronary Calcification and Stent

**DOI:** 10.3390/diagnostics16152408

**Published:** 2026-07-31

**Authors:** Aaron So, Satoshi Nakamura, Masafumi Takafuji, Mustafa Haider, Christian Rogers, Patrick Teefy, Kakuya Kitagawa

**Affiliations:** 1 Imaging Program, Lawson Research Institute, London, ON N6A 4V2, Canada; 2 Department of Medical Biophysics, Western University, London, ON N6A 5C1, Canada; 3 Department of Radiology, Mie University Hospital, Tsu 514-8507, Japan; 4 Division of Cardiology, London Health Sciences Centre, London, ON N6A 5A5, Canada

**Keywords:** fractional flow reserve, dynamic CT myocardial perfusion imaging, coronary artery disease, coronary calcification, stent

## Abstract

**Background/Objectives**: A novel CT dynamic angiographic imaging (CT-DAI) analytic algorithm was evaluated against the clinical gold standard for fractional flow reserve (FFR) measurement in patients with coronary artery disease (CAD) characterized by diffuse dense calcification and previous stent implantation. **Methods**: This retrospective feasibility study included 24 coronary arteries in 16 patients (age 69.9 ± 8.9 years, 11 males) with CAD who underwent dynamic CT myocardial perfusion scanning using a dual-source CT scanner after intravenous infusion of adenosine triphosphate. The included patients had analyzable proximal and distal coronary artery segments adjacent to the stenosis in the myocardial perfusion images and had corresponding invasive catheter-based FFR measurements for that stenosis. An in-house software based on the CT-DAI algorithm was used to compute FFR using the coronary time-enhancement curves sampled across the stenosis from stress myocardial CT perfusion images. The CT-DAI derived FFR values were then compared to the corresponding catheter-based FFR values. A coronary stenosis was considered functionally significant for FFR values below 0.8. **Results**: The mean axial length and calcium score of the coronary stenoses were 47.9 mm and 1911.63 Agatston Units, respectively. Eight coronary arteries received stents from previous treatments. The CT-DAI derived FFR values (0.822 ± 0.143) showed an excellent linear correlation (R = 0.974) with and were indifferent from the invasive FFR values (0.826 ± 0.147, *p* = 0.537), resulting in 100% per-vessel and per-patient sensitivity and specificity for the detection of functionally significant coronary stenosis. Bland–Altman analysis revealed a minimal mean difference in FFR measurements (0.004) between the two modalities with the lower and upper limits of agreement at −0.061 and 0.069, respectively. **Conclusions**: The findings suggest that CT-DAI can derive FFR for the coronary arteries with heavy calcification and stents from dynamic myocardial CT perfusion images.

## 1. Introduction

Fractional flow reserve (FFR), defined as the ratio of maximum coronary blood flow measured distal to a coronary stenosis to the normal maximum flow during hyperemia, is the gold standard for assessing the functional significance of coronary artery disease (CAD) [1,2]. Traditionally, FFR is measured by inserting a pressure wire across a coronary stenosis during cardiac catheterization. In recent years, the use of computed tomography-based FFR assessment (CT-FFR) as an alternative to catheter-based FFR has increased due to its non-invasiveness and its ability to reduce unnecessary invasive catheterizations [3]. The original CT-FFR technology is based on computational fluid dynamics derived from coronary CT angiography (CCTA) images [4]. However, previous meta-analyses have indicated that the diagnostic accuracy of CT-FFR for intermediate coronary stenoses is moderate [5,6]. As the underlying physiological model of CT-FFR relies on population data [7], assumptions about an individual’s specific response to hyperemic stimuli can be inaccurate, leading to significant variation in diagnostic accuracy across the spectrum of disease. Furthermore, the accuracy of CT-FFR depends on the precision of the three-dimensional coronary artery model generated from CCTA images [8,9]. Extensive coronary calcification can cause severe blooming artifacts [10], which obscure lumen evaluation and negatively affect the accuracy of the anatomical model used for fluid dynamics simulation [11,12]. Suboptimal diagnostic accuracy resulting from dense calcification has also been reported in the CT-FFR measurements based on the more recent machine learning approach [13,14].

We have introduced a novel CT dynamic angiographic imaging (CT-DAI) analytic algorithm for FFR measurement using dynamic CT myocardial perfusion images [15]. This approach may provide solutions for the current limitations of CT-FFR. Since many patients with CAD have concomitant epicardial and microvascular dysfunction, the additional FFR measurement obtained from CT perfusion images may be useful for tailoring treatment in patients with such complex conditions. Fundamentally, this analytic approach utilizes dynamic image data proximal and distal to a coronary stenosis to estimate the patient’s coronary blood flow and pressure, which in turn leads to the observed tracer kinetics across the lesion. In a previous retrospective feasibility study, this approach exhibited an excellent agreement with catheter-based FFR measurement for functional evaluation of CAD. In this study, we further investigated the feasibility of this approach for assessing functionally significant CAD in patients with diffuse calcified lesions and implanted stents.

## 2. Methods

### 2.1. Patient Cohort

**Figure 1:**
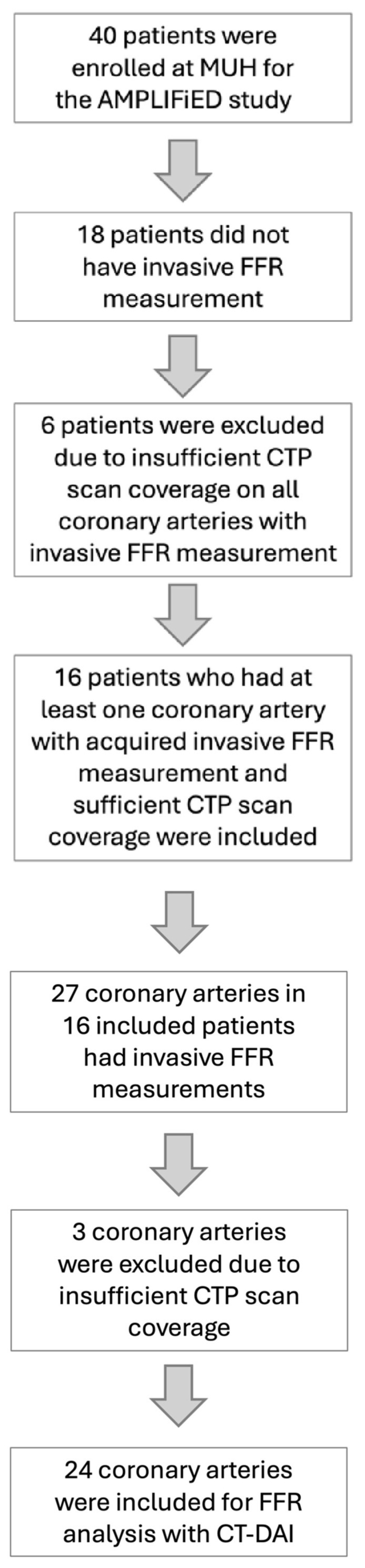
Patient and coronary artery inclusion flow chart [16]. Abbreviation: MUH = Mie University Hospital.

We retrospectively reviewed the clinical and CT image data acquired at one center (Mie University Hospital, Tsu, Japan) for the AMPLIFiED (Assessment of Myocardial Perfusion Linked to Infarction and Fibrosis Explored with Dual-Source CT) multicenter observational study [16]. A total of 16 patients were selected from the 40 available patients in this cohort for FFR analysis using CT-DAI. All of the selected cases satisfied the following criteria (Figure 1): (1) the dynamic CT myocardial perfusion scans sufficiently covered the proximal and distal coronary segments across the stenosis of interest. The distal coronary segment was at least one centimeter beyond the stenosis; (2) had invasive (catheter-based) coronary angiography of the artery where the stenosis of interest was located; (3) underwent invasive FFR measurement for the stenosis of interest; and (4) cardiac catheterization was performed within 60 days after stress (hyperemic) dynamic CT myocardial perfusion imaging.

### 2.2. CT Image Acquisition

Cardiac CT examinations were performed using a second-generation (Somatom Definition Flash) and a third-generation (Somatom Force) dual-source CT scanner (Siemens Healthineers, Erlangen, Germany). For each patient, dynamic myocardial CT perfusion imaging was initiated approximately three minutes into an intravenous infusion of 20 mg of adenosine triphosphate (ATP) at a rate of 160 µg/kg/min. The contrast administration protocol used for perfusion imaging was 40 mL of iopamidol (370 mgI/mL; Isovue, Bracco, Milan, Italy) with an injection rate of 5 mL/s, followed by a 40 mL saline flush at the same injection rate. Dynamic contrast-enhanced (perfusion) images of the heart were acquired at the end-systolic phases (250 msec after the R wave) for approximately 30 s with breath hold using an axial shuttle scan mode with the following scan settings: collimation = 32 × 1.2 (Definition Flash) or 48 × 1.2 mm (Force), gantry rotation period = 0.28 or 0.25 s, tube voltage = 80 or 70 kV, tube current = 300 mA⋅s per gantry rotation. The axial scan coverage associated with a second and a third-generation dual-source scanner was 7.3 and 10.5 cm, respectively. The ATP infusion was stopped immediately after the perfusion scan was completed.

Ten minutes later, sublingual nitroglycerin was administered, and iopamidol was injected at 26 mgI/kg/s over 12 s before a standard CCTA was acquired. The patient’s heart rate was controlled with an intravenous injection of landiolol hydrochloride (Corebeta, Ono Pharmaceutical Co., Ltd., Osaka, Japan) before CCTA acquisition, if required, aiming for a heart rate below 60 beats per minute. The acquisition parameters of CCTA with a second- or third-generation dual-source scanner were: collimation = 128 × 0.6 or 192 × 0.6 mm and gantry rotation period = 0.28 or 0.25 s, respectively. The scanners automatically determined the optimal tube voltage and current settings for the CCTA acquisition. The axial CCTA and CT perfusion images were reconstructed using a smooth kernel (I26 or Br40) and an iterative reconstruction algorithm (Strength 3, SAFIRE or ADMIRE, Siemens Healthineers) [17,18] with slice thicknesses of 0.6 mm and 2 mm, respectively.

### 2.3. Invasive Coronary Angiography and FFR Procedures

Invasive coronary angiography was performed according to standard techniques within 60 days of the cardiac CT examination. During the procedure, the coronary arteries were visually assessed. If a diameter stenosis of 26% to 90% was visually estimated in the right coronary artery (RCA), left anterior descending artery (LAD), left circumflex artery (LCX), or their significant branches (≥1.5 mm in diameter), FFR was measured using a sensor-tipped guidewire as far as the procedure deemed safe. The FFR value was calculated as the ratio between the mean coronary artery pressure distal to the coronary stenosis measured by the pressure wire and the mean aortic pressure measured through the guiding catheter, recorded simultaneously under conditions of maximal hyperemia induced by a continuous intravenous infusion of ATP (0.16 mg/kg/min for a minimum of 3 min).

### 2.4. CT Image Processing

#### 2.4.1. Correction of Residual Motion

The myocardial CT perfusion images were transferred to a GE Advantage Window Workstation for motion correction. The images underwent image registration using a 3D non-rigid image registration algorithm (CT Perfusion 4D, GE Healthcare, Waukesha, WI, USA) to minimize residual cardiac and respiratory motion [19].

#### 2.4.2. CT-DAI Analytic Algorithm

The registered perfusion images were transferred to a desktop computer for FFR analysis using an in-house software developed with MATLAB (Version 2023a, The MathWorks, Inc., Natick, MA, USA). The analytic algorithm is described in the Supplementary Materials Section.

### 2.5. Sampling of Coronary Time-Enhancement Curves

#### 2.5.1. Pre-Lesion and Post-Lesion Curves

**Figure 2:**
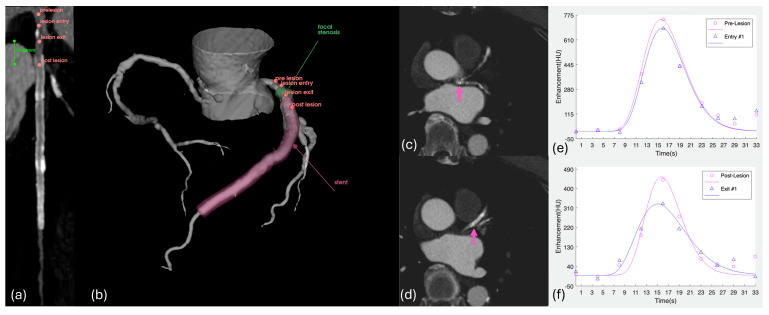
A 74-year-old female (patient 11 in Table 1) whose left anterior descending artery (LAD) had a 25% focal stenosis in the proximal segment followed by a stent that extended to the distal segment. The FFR value measured in the mid-LAD segment (coronary segment #7) had a CT-DAI of 0.85, which was close to the value of 0.86 acquired with the invasive catheter-based method. (**a**) Straightened lumen view of the LAD with the sampling regions of interest (ROIs) at the pre-lesion, lesion entry, lesion exit and post-lesion sites. The post-lesion ROI was placed within the stent in the mid-LAD segment to match the invasive FFR measurement site. (**b**) Sampling ROIs shown in the three-dimensional image of the LAD. (**c**) Pre-lesion ROI (pink arrow) and (**d**) post-lesion ROI (pink arrow) shown in the axial CT myocardial perfusion image at one time point. (**e**,**f**) The plots show the raw data points sampled from the ROIs shown in (**a**–**d**) and the corresponding fitted coronary time-enhancement curves.

In each patient, non-invasive FFR measurement using CT-DAI was performed in the same coronary artery segment [20] as invasive FFR measurement. The post-lesion coronary time-enhancement curve was usually sampled approximately 1 cm beyond from the exit side of the stenosis with acquired invasive FFR [21]. If a stent was present immediately distal to the invasively investigated stenosis, the post-lesion coronary time-enhancement curve would be sampled approximately 1 cm beyond the distal end of the stent, taking care to be within the same coronary artery segment where invasive FFR was acquired. An exceptional case was patient 11 in Table 1 and Figure 2, where CT FFR measurement was acquired in the stented mid-LAD segment to match the corresponding invasive FFR measurement. In situations where a coronary artery segment had acquired invasive FFR but without stenosis or stent, the CT-derived post-lesion coronary time-enhancement curve would be sampled in the middle of the segment.

If the stenosis of interest resided in the RCA, the pre-lesion coronary time-enhancement curve would be sampled in the proximal RCA. If the stenosis of interest was in a left coronary artery, the pre-lesion curve would be sampled in the left main or in the proximal LAD or LCX segment.

#### 2.5.2. Lesion Entry and Exit Curves

**Figure 3:**
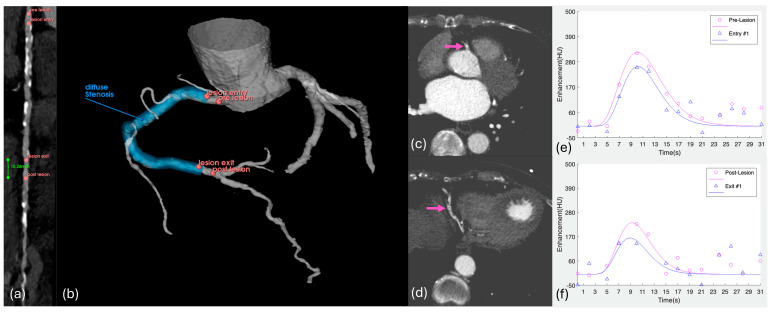
A 72-year-old female (patient 16 in Table 1) whose right coronary artery (RCA) had a long 75% diffuse stenosis that extended from the proximal to distal segment. The FFR value measured in the distal segment (coronary segment #3) with CT-DAI was 0.83 and agreed with that obtained using the invasive method (0.83). (**a**) Pre-lesion, lesion entry, lesion exit and post-lesion sampling ROIs shown in the straightened lumen view of the RCA. The calcified plaques below the post-lesion site were not included for the FFR analysis because the post-lesion ROI would have been placed in coronary segment #4. (**b**) Sampling ROIs shown in the three-dimensional image of the RCA. (**c**) Pre-lesion ROI (pink arrow) and (**d**) post-lesion ROI (pink arrow) shown in the axial CT myocardial perfusion image at one time point. (**e**,**f**) The plots show the raw data points sampled from the ROIs shown in (**a**–**d**) and the corresponding fitted coronary time-enhancement curves.

**Figure 4:**
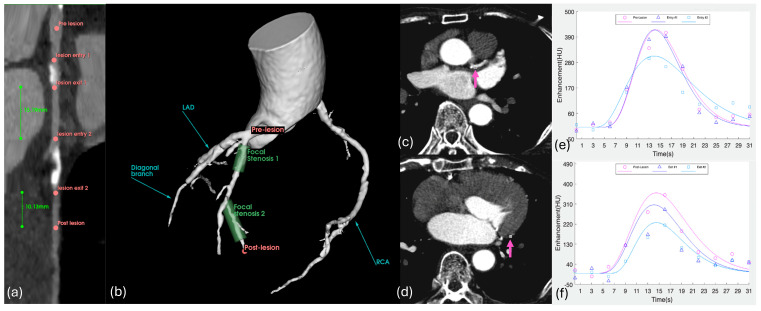
An 80-year-old male (patient 14 in Table 1) whose left circumflex artery (LCX) had two 75% focal stenoses apart from each other by 1.5 cm. The FFR value measured with CT- DAI below the second stenosis in the distal LCX segment (coronary segment #13) was 0.87. In comparison, the FFR value acquired with the invasive method in the same coronary segment was 0.90. (**a**) Straightened lumen view of the LCX with the sampling ROIs. Two pairs of lesion entry and exit ROIs were placed between the pre-lesion and post-lesion ROIs. (**b**) Locations of the stenoses and sampling ROIs shown in the three-dimensional image of the LCX. (**c**) Pre-lesion ROI (pink arrow) and (**d**) post-lesion ROI (pink arrow) shown in the axial CT myocardial perfusion image at one time point. (**e**,**f**) The plots reveal the raw data points sampled from the ROIs shown in (**a**–**d**) and the corresponding fitted coronary time-enhancement curves.

The coronary time-enhancement curves adjacent to the entry and exit sides of a stenosis were sampled to compute the pressure loss arising from abrupt lumen restriction and expansion associated with the stenosis (see Supplementary Materials). In situations where multiple stenoses were present between the selected pre- and post-lesion sampling sites, the number of entry and exit coronary time-enhancement curves required for FFR computation with CT-DAI would depend on the classifications of the stenoses. A stenosis shorter or longer than 20 mm would be considered a focal or diffuse stenosis [22], respectively, with the latter clinical manifestation illustrated in Figure 3. If two focal stenoses were separated by more than 10 mm, they would be considered two independent serial stenoses [22], and a pair of entry and exit time-enhancement curves would be sampled for each stenosis (a total of two pairs of entry/exit curves would be needed, see Figure 4). On the contrary, if adjacent focal stenoses were separated by less than 10 mm, they would be considered part of a long diffuse stenosis and only one pair of entry and exit time-enhancement curves would be needed.

### 2.6. CT Perfusion Analysis

Dynamic contrast-enhanced images were also analyzed using a commercial CT perfusion analysis software (Syngo.via, Siemens Healthineers), and the results have been published previously [16]. In brief, this software utilizes a hybrid deconvolution and maximum-slope analytic algorithm to generate voxel-based myocardial blood flow (MBF) maps [23]. Regions of interest were manually placed within each of the 16 short-axis myocardial segments [24] on the MBF maps to get the absolute segmental MBF values in units of mL/min/100 g. The absolute MBF value in each short-axis myocardial segment within the same coronary territory (LAD, LCX, or RCA) was compared, and the minimum value was used to represent the absolute MBF for that coronary territory. The relative MBF value for each coronary territory was also computed by dividing the selected absolute MBF by the global maximum MBF value of the 16 short-axis myocardial segments.

### 2.7. Comparison with Invasive FFR and CT Perfusion Measurements

Statistical analyses were performed using the SPSS software (version 30.0; IBM, Armonk, NY, USA). FFR measurements obtained with CT-DAI and pressure wires were compared using linear regression analysis to assess whether the two measurement sets were linearly correlated. These measurements were also evaluated using the Bland–Altman analysis to determine the limits of agreement between the two modalities. The mean of the FFR values acquired with each modality was also compared using a two-sided paired *t*-test to determine if the difference was statistically significant. The per-vessel and per-patient diagnostic performance (accuracy, sensitivity, specificity, positive predictive value, and negative predictive value) of CT perfusion and CT-DAI for detecting functionally significant CAD in this patient cohort were evaluated using binary logistic regression analysis with invasive FFR measurement as the gold standard. The 95% confidence intervals of the performance metrics associated with CT-DAI were estimated using the Wilson score interval formula [25].

## 3. Results

### 3.1. Patients

A total of 16 patients (Male = 11; Female = 5; average age = 69.9 years) met the inclusion criteria and were included in this retrospective study. The clinical and demographic details of these patients are summarized in Table 1. Eleven patients underwent cardiac CT scans using a 3rd-generation dual-source CT scanner, while five patients had examinations with a 2nd-generation dual-source CT scanner. The mean of the effective doses of the dynamic myocardial perfusion scans, also provided in Table 1, was 4.1 ± 0.8 mSv. Half of the patients (8/16) had a coronary calcium scan, with a mean calcium score of 1911.63 Agatston Units (AU). For most of the remaining patients, calcification in the coronary arteries was visibly extensive across multiple tomographic slices. Eight coronary arteries in six patients had previously received stents during PCI procedures. According to the image analysis protocol described in Section 2, all of the 24 coronary arteries with invasive FFR measurements had at least a single stenosis between the pre- and post-lesion sampling sites, and six of them had two stenoses between the pre- and post-lesion sites. The mean axial length of the first stenosis (closer to the proximal coronary segment) was 47.9 mm (8 focal and 16 diffuse; 10.1 mm and 66.8 mm, respectively). The mean axial length of the second stenosis (closer to the distal coronary segment) was 17.1 mm (3 focal and 3 diffuse; 6.0 mm and 32.0 mm, respectively).

Compared to our previous pilot study on Korean patients [15], the Japanese patients in the current study exhibited denser coronary calcification and more diffuse coronary disease. Thirteen patients (13/14) in the previous study had a coronary calcium score, with a mean score of 149.58 AU. Additionally, the mean axial length of the stenosis was 0.84 mm, and only one stenosis was present between the pre- and post-lesion sampling locations in all Korean patients.

#### 3.1.1. Comparison in FFR Measurement Between CT-DAI and Invasive Methods

**Figure 5:**
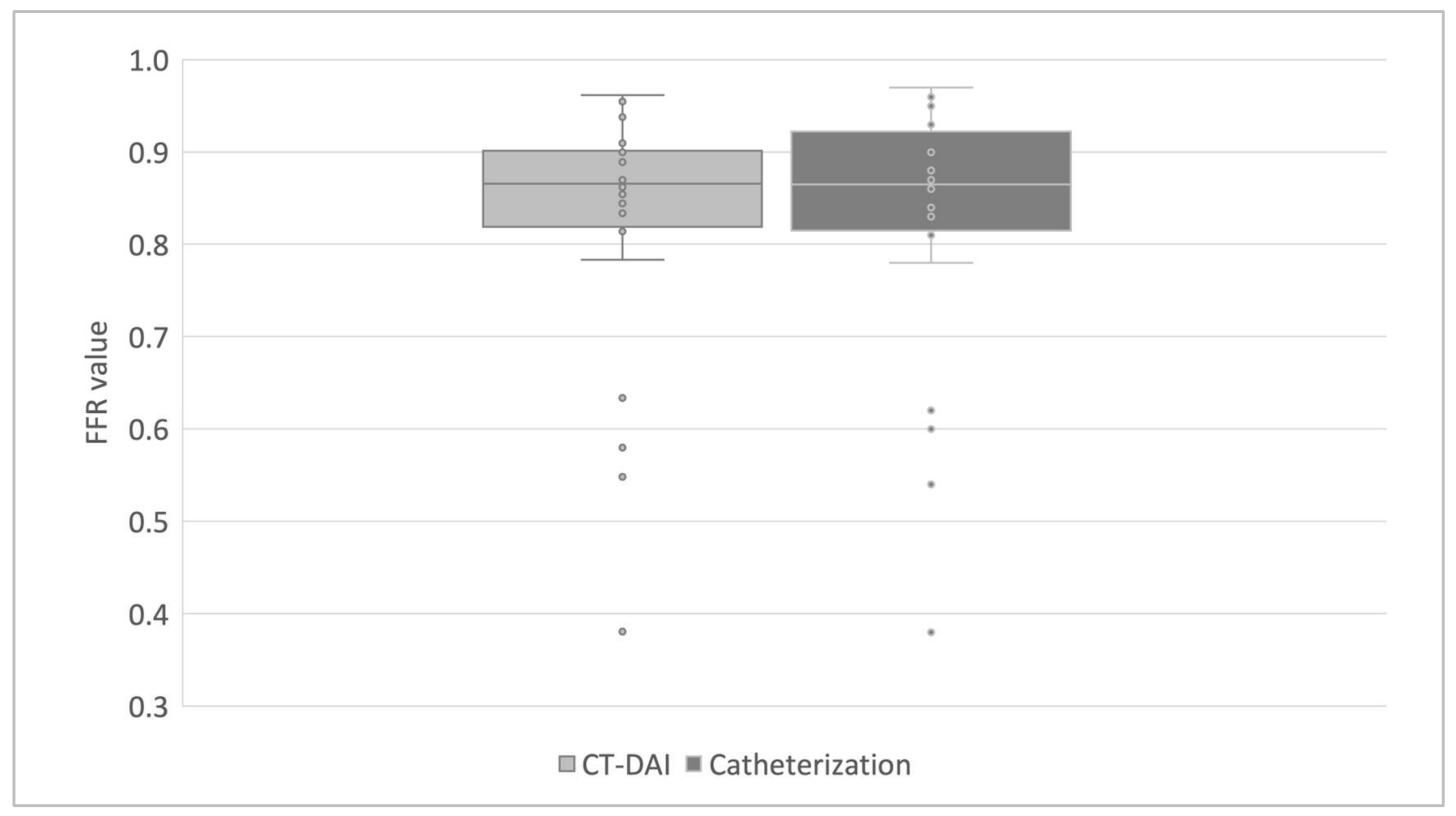
Box plot of the FFR values measured with the CT-DAI and invasive catheterization methods. The upper and lower boundaries of each box represent the upper and lower quartiles of the FFR values measured by the corresponding method. The horizontal line within each box represents the median FFR value measured by the corresponding method. The upper and lower whiskers of each box represent the maximum and minimum FFR values measured by the corresponding method. The circles in each group represent the outlier FFR values.

**Figure 6:**
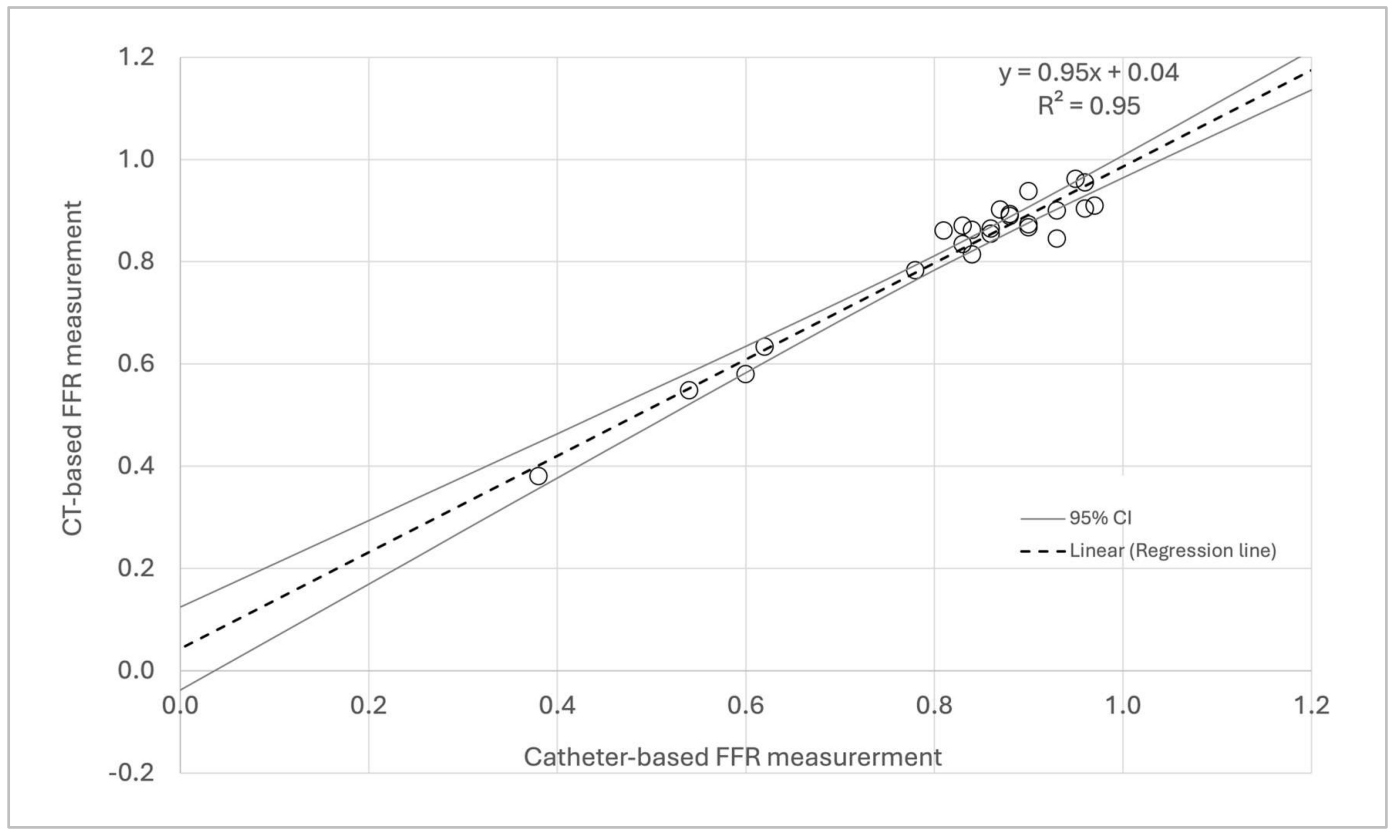
Linear regression plot of the FFR values measured with the CT-DAI and invasive methods. The correlation coefficient (R) between the two sets of measurement was 0.975.

**Figure 7:**
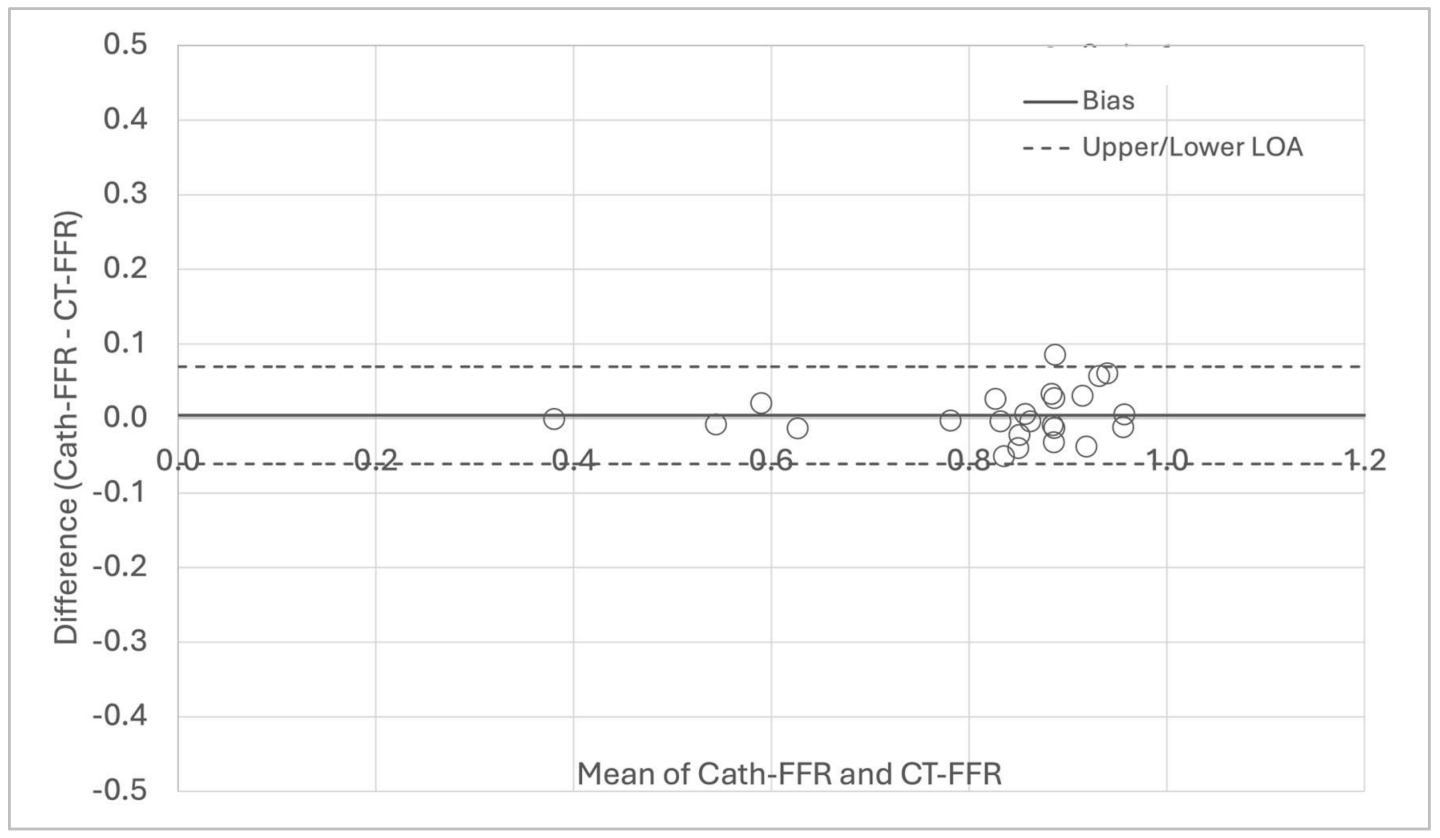
Blant-Altman plot for the CT-DAI and catheter-based FFR measurements. The mean bias of CT-DAI relative to the catheter-based method and the limits of agreement is shown in the graph. Abbreviations: CI = confidence interval; LOA = limits of agreement.

A total of 24 coronary arteries from the 16 patients had invasive FFR measurement and met the inclusion criteria for this feasibility study (Table 1). Based on a cutoff of 0.8, invasive FFR assessment determined that 5 coronary stenoses were functionally significant. The mean and standard deviation of FFR measured in the 24 coronary arteries with CT-DAI were 0.822 and 0.143, respectively, and not statistically different (*p* = 0.537) from the values corresponding to the invasive catheterization method (mean = 0.826, standard deviation = 0.147; Figure 5). The linear regression analysis revealed a correlation coefficient of 0.974 between the two sets of FFR measurements (*p* < 0.001; Figure 6). The slope and intercept of the regression line were 0.95 and 0.04, respectively. The Bland–Altman analysis showed no significant proportional bias in the FFR measurements with CT-DAI across the spectrum of disease (Figure 7). On average, CT-DAI-derived FFR was lower than catheter-derived FFR by 0.004, with the lower and upper limits of agreement at −0.061 and 0.069, respectively.

#### 3.1.2. Comparison in Diagnostic Performance Between CT-DAI and CT Perfusion

Invasive FFR measurements identified that 5 of the 24 coronary arteries (3 patients) had functionally significant stenosis. The binary logistic regression analysis revealed that the optimal threshold for the FFR measured with CT-DAI was 0.80, differentiating between functionally significant and non-significant coronary stenoses in keeping with the invasive FFR threshold. With this optimal threshold, CT-DAI demonstrated 100% sensitivity, specificity, positive predictive value, and negative predictive value in identifying functionally significant CAD in both per-vessel and per-patient analyses (Table 2).

Two sets of absolute and relative MBF thresholds were used for the comparison between CT perfusion and CT FFR measurements. One set of MBF thresholds was previously determined from all patient data available for the AMPLIFiED study (absolute MBF = 116 mL/min/100 g and relative MBF = 0.71) [16,26]. Another set of MBF thresholds was derived from a binary logistic regression analysis on the available patient data for this retrospective analysis. The diagnostic performance of CT-DAI was superior to CT myocardial perfusion for functional assessment of CAD with respect to invasive FFR measurement in both per-vessel and per-patient analyses, regardless of the choice of MBF thresholds (Table 2).

## 4. Discussion

This study aims to evaluate the feasibility of CT-DAI for functional assessment in real-world clinical manifestations of CAD, compared with gold standard invasive FFR measurement. The main findings of this study align with those of the previous feasibility study: CT-DAI is accurate for detecting functionally significant epicardial coronary stenoses in highly controlled CT myocardial perfusion cases. The narrow 95% confidence interval of the mean regression line (Figure 6) suggests that the linear relationship between the two modalities is reproducible.

Substantial differences were observed between the patients in the current study and those in the preceding investigation. Firstly, the present patients exhibited denser calcification within the coronary arteries, as indicated by a markedly higher mean coronary calcium score. Secondly, the current cohort demonstrated more diffuse coronary disease and a greater number of implanted stents. The findings of this study suggest that the CT-DAI algorithm effectively manages various coronary conditions, thereby yielding comparable diagnostic performance across both studies and demonstrating reliability in advanced calcification and diffuse atherosclerosis, both of which are commonly characterized in patients with CAD.

Two sets of absolute and relative MBF thresholds were used for the comparison between CT perfusion and CT FFR measurements because the original MBF thresholds were determined from a larger patient cohort that was not fully accessible for this study. As such, redefining the MBF thresholds for the subset of patients available for this study using logistic regression was necessary, and this approach was proven to be correct. For instance, the new threshold (0.43) increased the diagnostic accuracy of relative MBF for functional CAD assessment in this smaller cohort from 70.8% to 83.3%. The suboptimal accuracy of CT perfusion for functional assessment of CAD, as also confirmed by previous studies [25], could be attributed to the presence of microvascular disease in some of the patients [27,28,29]. The results of our first two retrospective studies suggest that the concomitant use of myocardial CT perfusion and CT FFR measurement may improve the overall functional diagnosis of CAD from a dynamic contrast-enhanced CT scan.

This investigation also demonstrates the capability of CT-DAI for functional assessment in stented coronary arteries. Baseline voxel intensity within a stented segment could be above 500 Hounsfield Units (HU), markedly higher than native blood (~50 HU), primarily due to blooming artifacts from the metallic stent [10]. As depicted in Figure 2, by applying appropriate baseline subtraction, an accurate coronary time-enhancement curve could still be generated for FFR estimation. Recent advances in image processing software and scanner hardware development (such as photon counting CT [30]) that improve stented lumen visualization [31] may facilitate the clinical adoption of this new analytic approach for FFR measurement.

It has been suggested that the diagnostic accuracy of CT-FFR is lower in moderately stenosed coronary arteries than that in severely stenosed and non-stenosed coronary arteries [5,6]. In the current and preceding [15] retrospective feasibility studies, more than half of the coronary arteries included for analysis had an intermediate degree of stenosis (50–75%), and the accuracy of CT-DAI derived FFR remained high. Our findings suggest that the temporal information acquired across a stenosis from dynamic myocardial CT perfusion images may enable a more realistic estimation of the pressure loss induced by the stenosis compared to fluid dynamics simulations based on anatomical information obtained from CCTA images. Moreover, this study presents a robust analysis protocol for rapid FFR assessment in coronary arteries with stents or multiple stenoses. Classifying stenoses as focal, diffuse, or serial—based on their length and proximity to other stenoses—guides the number of entry/exit time-enhancement curves needed for FFR measurement with CT-DAI.

Several limitations are acknowledged in this study. First, the analysis was limited to 16 patients and 24 coronary arteries, of which only five were functionally significant. Since the second-generation dual-source CT scanner was not used in all of the myocardial CT perfusion studies available for this study, some coronary arteries with invasive FFR measurements were excluded as a result of inadequate scan coverage. Thus, the diagnostic performance metrics derived from this limited feasibility study must be interpreted with caution. Pivotal clinical validation studies are necessary to determine whether CT-DAI can provide additional diagnostic value beyond CCTA for the detection of hemodynamically significant CAD, comparable to other existing CT-based methods [32], specifically in cases of dense coronary calcification where the current CT-FFR approach could be less accurate [33]. Second, the CT-DAI analysis was not blinded to the invasive FFR results due to uncertainty about the optimal post-lesion sampling location for agreement with catheter-based FFR measurements. Our results indicated that the FFR measurements acquired at approximately 1 cm distal to the stenosis were highly accurate, consistent with prior findings [15,21]. Future larger clinical validation studies should be conducted with blinding to the gold standard after comprehensive testing of the CT-DAI algorithm with smaller feasibility studies. Third, this study did not examine intra- and inter-observer variability, as our ongoing technical development aims to automate the pre- and post-lesion sampling processing to minimize inconsistences caused by human input. Fourth, the interval between the CT perfusion scan and catheterization was as long as 60 days, and lesion physiology could change during this time, making comparisons between the two modalities less reliable.

In summary, this early feasibility study demonstrated that the CT-DAI analytic algorithm can measure FFR in stented and diffusely calcified coronary arteries from dynamic myocardial CT perfusion images. A larger, blinded, prospective multicenter validation is needed to conclude whether this technical advancement can improve the accuracy of CT-based functional diagnosis of CAD.

## Figures and Tables

**Table 1:** Clinical and demographic information of the patients included in the study, and the radiation dose of the stress CT myocardial perfusion scans performed on the patients. Abbreviations: MI = myocardial infarction; PCI = percutaneous coronary intervention; ICA = invasive coronary angiography; RCA = right coronary artery; LAD = left anterior descending artery; LCX = left circumflex artery; DLP = dose-length product. Cells filled with solid grey color represent the coronary arteries with invasive FFR measurement and included for FFR analysis with CT-DAI. Cells filled with grey stripes represent the coronary arteries with invasive FFR measurement but excluded for FFR analysis with CT-DAI due to insufficient CTP scan coverage. For patients 1 to 6, the perfusion scan was obtained using a second-generation dual-source CT scanner, which provides 7.3 cm axial scan coverage through axial shuttle acquisition. For patients 7 to 16, the scan was performed with a third-generation dual-source CT scanner, offering 10.5 cm axial scan coverage via the same axial shuttle approach.

Patient Number	Sex	Age (Year)	Height (cm)	Weight (kg)	Body Mass Index (kg/m^2^)	Calcium Score	Prior MI	Prior PCI	Degree of Stenosis Assessed by ICA			DLP (mGy·cm)	Effective Dose (mSv)
									RCA	LAD	LCX		
1	M	65	160.5	104	40.4	-	N	Y	25	100	75	328	4.6
2	M	83	152	51	22.1	-	N	N	50	90	90	290	4.1
3	F	69	161	57	22.0	110	N	N	75	25	25	283	4.0
4	F	79	147	55	25.5	253	N	N	25	50	-	446.9	6.3
5	M	76	154.3	51	21.4	1022	N	N	25	-	90	243.8	3.4
6	F	75	155.3	60	24.9	-	Y	Y	75	90	50	270.3	3.8
7	M	53	170.8	67.4	23.1	-	Y	Y	-	25	75	263.6	3.7
8	M	59	171.1	83	28.4	856	N	N	0	0	0	319.5	4.5
9	M	61	165.8	73	26.6	-	Y	Y	50	75	25	366.9	5.1
10	M	60	163.7	61.7	23.0	-	Y	Y	90	25	50	218.1	3.1
11	F	74	153.4	51	21.7	-	N	N	50	25	50	204	2.9
12	M	61	175	81	26.4	-	N	Y	25	50	-	322.6	4.5
13	M	74	160.1	58.5	22.8	1523	N	N	25	75	-	302.9	4.2
14	M	80	165.8	49.5	18.0	2771	N	N	25	50	75	223.3	3.1
15	M	77	160	52.9	20.7	3141	N	N	90	50	100	278.3	3.9
16	F	72	151.8	51	22.1	5617	Y	N	75	90	99	279.1	3.9

**Table 2:** Comparison of coronary artery disease (CAD) functional assessment between MBF measurement with CT perfusion and FFR measurement with CT-DAI. “Threshold 1” represents the optimal cutoff values of absolute MBF (116 mL/min/100 g) and relative MBF (0.71) determined from a previous prospective multicenter study [16]. “Threshold 2” denotes the optimal cutoff values of absolute MBF (41 mL/min/100 g) and relative MBF (0.43) determined from a binary logistic regression analysis on the data available for this study. Abbreviations: MBF = myocardial blood flow; FFR = fractional flow reserve; CT-DAI = CT dynamic angiographic imaging analytic algorithm.

	Per-Vessel Analysis					Per-Patient Analysis				
	Absolute MBF (Cutoff 1)	Relative MBF (Cutoff 1)	Absolute MBF (Cutoff 2)	Relative MBF (Cutoff 2)	CT-DAI- Derived FFR	Absolute MBF (Cutoff 1)	Relative MBF (Cutoff 1)	Absolute MBF (Cutoff 2)	Relative MBF (Cutoff 2)	CT-DAI- Derived FFR
Sensitivity	100%	100%	20%	40%	100% (86.2–100%)	100%	100%	33.3%	66.7%	100% (86.2–100%)
Specificity	26.3%	63.2%	100%	94.7%	100% (86.2–100%)	15.4%	53.8%	100%	92.3%	100% (86.2–100%)
PPV	26.3%	41.7%	100%	66.7%	100% (86.2–100%)	21.5%	33.3%	100%	67%	100% (86.2–100%)
NPV	100%	100%	82.6%	85.7%	100% (86.2–100%)	100%	100%	86.7%	92.3%	100% (86.2–100%)
Accuracy	41.7%	70.8%	83.3%	83.3%	100% (86.2–100%)	31.3%	62.5%	87.5%	87.5%	100% (86.2–100%)

## Data Availability

The data presented in this study are available on request from the corresponding author.

## Supplementary Materials

The following supporting information can be downloaded at https://www.mdpi.com/article/10.3390/diagnostics16152408/s1. References [34,35,36,37] are cited in the supplementary materials.

## References

[1] Pijls N.H., Van Gelder B., Van der Voort P., Peels K., Bracke F.A., Bonnier H.J., el Gamal M.I. (1995). Fractional flow reserve. A useful index to evaluate the influence of an epicardial coronary stenosis on myocardial blood flow. Circulation.

[2] Tonino P.A.L., De Bruyne B., Pijls N.H.J., Siebert U., Ikeno F., van’t Veer M., Klauss V., Manoharan G., Engstrøm T., Oldroyd K.G. (2009). FAME Study Investigators. Fractional flow reserve versus angiography for guiding percutaneous coronary intervention. N. Engl. J. Med..

[3] Rajiah P., Cummings K.W., Williamson E., Young P.M. (2022). CT Fractional Flow Reserve: A Practical Guide to Application, Interpretation, and Problem Solving. Radiographics.

[4] Min J.K., Taylor C.A., Achenbach S., Koo B.K., Leipsic J., Nørgaard B.L., Pijls N.J., De Bruyne B. (2015). Noninvasive Fractional Flow Reserve Derived From Coronary CT Angiography: Clinical Data and Scientific Principles. JACC Cardiovasc. Imaging.

[5] Cook C.M., Petraco R., Shun-Shin M.J., Ahmad Y., Nijjer S., Al-Lamee R., Kikuta Y., Shiono Y., Mayet J., Francis D.P. (2017). Diagnostic Accuracy of Computed Tomography-Derived Fractional Flow Reserve: A Systematic Review. JAMA Cardiol..

[6] Faulder T.I., Prematunga K., Moloi S.B., Faulder L.E., Jones R., Moxon J.V. (2024). Agreement of Fractional Flow Reserve Estimated by Computed Tomography With Invasively Measured Fractional Flow Reserve: A Systematic Review and Meta-Analysis. J. Am. Heart Assoc..

[7] Lo E.W., Menezes L.J., Torii R. (2020). On outflow boundary conditions for CT-based computation of FFR: Examination using PET images. Med. Eng. Phys..

[8] Kim H.J., Vignon-Clementel I.E., Coogan J.S., Figueroa C.A., Jansen K.E., Taylor C.A. (2010). Patient-specific modeling of blood flow and pressure in human coronary arteries. Ann. Biomed. Eng..

[9] He Y., Northrup H., Le H., Cheung A.K., Berceli S.A., Shiu Y.T. (2022). Medical Image-Based Computational Fluid Dynamics and Fluid-Structure Interaction Analysis in Vascular Diseases. Rev. Front. Bioeng. Biotechnol..

[10] Pack J.D., Xu M., Wang G., Baskaran L., Min J., De Man B. (2022). Cardiac CT blooming artifacts: Clinical significance, root causes and potential solutions. Vis. Comput. Ind. Biomed. Art..

[11] Mickley H., Veien K.T., Gerke O., Lambrechtsen J., Rohold A., Steffensen F.H., Husic M., Akkan D., Busk M., Jessen L.B. (2022). Diagnostic and Clinical Value of FFRCT in Stable Chest Pain Patients With Extensive Coronary Calcification: The FACC Study. JACC Cardiovasc. Imaging.

[12] Nørgaard B.L., Leipsic J., Gaur S., Seneviratne S., Ko B.S., Ito H., Jensen J.M., Mauri L., De Bruyne B., Bezerra H. (2014). NXT Trial Study Group. Diagnostic performance of noninvasive fractional flow reserve derived from coronary computed tomography angiography in suspected coronary artery disease: The NXT trial (Analysis of Coronary Blood Flow Using CT Angiography: Next Steps). J. Am. Coll. Cardiol..

[13] Itu L., Rapaka S., Passerini T., Georgescu B., Schwemmer C., Schoebinger M., Flohr T., Sharma P., Comaniciu D. (2016). A machine-learning approach for computation of fractional flow reserve from coronary computed tomography. J. Appl. Physiol..

[14] Tesche C., Otani K., De Cecco C.N., Coenen A., De Geer J., Kruk M., Kim Y.H., Albrecht M.H., Baumann S., Renker M. (2020). Influence of Coronary Calcium on Diagnostic Performance of Machine Learning CT-FFR: Results From MACHINE Registry. JACC Cardiovasc. Imaging.

[15] So A., Choo K.S., Lee J.W., Kim Y.H., Haider M., Hasan M., El-Ganga S., Goela A., Teefy P., Choe Y.H. (2024). Fractional flow reserve measurement using dynamic CT perfusion imaging in patients with coronary artery disease. Radiol. Adv..

[16] Kitagawa K., Nakamura S., Ota H., Ogawa R., Shizuka T., Kubo T., Yi Y., Ito T., Nagasawa N., Omori T. (2021). Diagnostic Performance of Dynamic Myocardial Perfusion Imaging Using Dual-Source Computed Tomography. J. Am. Coll. Cardiol..

[17] Chen B., Ramirez Giraldo J.C., Solomon J., Samei E. (2014). Evaluating iterative reconstruction performance in computed tomography. Med. Phys..

[18] Ellmann S., Kammerer F., Allmendinger T., Hammon M., Janka R., Lell M., Uder M., Kramer M. (2018). Advanced Modeled Iterative Reconstruction (ADMIRE) Facilitates Radiation Dose Reduction in Abdominal CT. Acad. Radiol..

[19] Chandler A., Wei W., Anderson E.F., Herron D.H., Ye Z., Ng C.S. (2012). Validation of motion correction techniques for liver CT perfusion studies. Br. J. Radiol..

[20] Austen W., Edwards J., Frye R., Gensini G.G., Gott V.L., Griffith L.S., McGoon D.C., Murphy M.L., Roe B.B. (1975). A Reporting System on Patients Evaluated for Coronary Artery Disease. Report of the Ad Hoc Committee for Grading of Coronary Artery Disease, Council on Cardiovascular Surgery, American Heart Association. Circulation.

[21] Nørgaard B.L., Fairbairn T.A., Safian R.D., Rabbat M.G., Ko B., Jensen J.M., Nieman K., Chinnaiyan K.M., Sand N.P., Matsuo H. (2019). Coronary CT angiography-derived fractional flow reserve testing in patients with stable coronary artery disease: Recommendations on interpretation and reporting. Radiol. Cardiothorac. Imaging.

[22] Scarsini R., Fezzi S., Leone A.M., De Maria G.L., Pighi M., Marcoli M., Tavella D., Pesarini G., Banning A.P., Barbato E. (2022). Functional Patterns of Coronary Disease: Diffuse, Focal, and Serial Lesions. JACC Cardiovasc. Interv..

[23] Mahnken A.H., Klotz E., Pietsch H., Schmidt B., Allmendinger T., Haberland U., Kalender W.A., Flohr T. (2010). Quantitative whole heart stress perfusion CT imaging as noninvasive assessment of hemodynamics in coronary artery stenosis: Preliminary animal experience. Investig. Radiol..

[24] Cerqueira M.D., Weissman N.J., Dilsizian V., Jacobs A.K., Kaul S., Laskey W.K., Pennell D.J., Rumberger J.A., Ryan T., Verani M.S. (2002). American Heart Association Writing Group on Myocardial Segmentation and Registration for Cardiac Imaging. Standardized myocardial segmentation and nomenclature for tomographic imaging of the heart. A statement for healthcare professionals from the Cardiac Imaging Committee of the Council on Clinical Cardiology of the American Heart Association. Circulation.

[25] Wei L., Hutson A.D. (2013). A comment on sample size calculations for binomial confidence intervals. J. Appl. Stat..

[26] Nagasawa N., Nakamura S., Ota H., Ogawa R., Nakashima H., Hatori N., Wang Y., Kurita T., Dohi K., Sakuma H. (2025). Relationship between microvascular status and diagnostic performance of stress dynamic CT perfusion imaging. Eur. Radiol..

[27] Ford T.J., Corcoran D., Berry C. (2017). Coronary artery disease: Physiology and prognosis. Eur. Heart J..

[28] Celeng C., Leiner T., Maurovich-Horvat P., Merkely B., de Jong P., Dankbaar J.W., van Es H.W., Ghoshhajra B.B., Hoffmann U., Takx R.A.P. (2019). Anatomical and Functional Computed Tomography for Diagnosing Hemodynamically Significant Coronary Artery Disease: A Meta-Analysis. JACC Cardiovasc. Imaging.

[29] Michallek F., Nakamura S., Takafuji M., Nagasawa N., Kurita T., Dohi K., Ota H., Nishimiya K., Ogawa R., Shizuka T. (2025). Fractal analysis of dynamic stress myocardial CT perfusion decouples diagnostic accuracy for obstructive coronary artery disease from remote flow. Jpn. J. Radiol..

[30] Hagar M.T., Soschynski M., Saffar R., Molina-Fuentes M.F., Weiss J., Rau A., Schuppert C., Ruile P., Faby S., Schibilsky D. (2024). Ultra-high-resolution photon-counting detector CT in evaluating coronary stent patency: A comparison to invasive coronary angiography. Eur. Radiol..

[31] Johnson N.P., Kirkeeide R.L., Gould K.L. (2012). Is Discordance of Coronary Flow Reserve and Fractional Flow Reserve Due to Methodology or Clinically Relevant Coronary Pathophysiology?. JACC Cardiovasc. Imag..

[32] Soschynski M., Storelli R., Birkemeyer C., Hagar M.T., Faby S., Schwemmer C., Nous F.M.A., Pugliese F., Vliegenthart R., Schlett C.L. (2024). CT Myocardial Perfusion and CT-FFR versus Invasive FFR for Hemodynamic Relevance of Coronary Artery Disease. Radiology.

[33] Ma Z., Tu C., Zhang B., Zhang D., Song X., Zhang H. (2024). A meta-analysis comparing the diagnostic performance of computed tomography-derived fractional flow reserve and coronary computed tomography angiography at different levels of coronary artery calcium score. Eur. Radiol..

[34] Klocke F.J., Greene D.G., Koberstein R.C. (1968). Indicator-Dilution Measurement of Cardiac Output with Dissolved Hydrogen. Circ. Res..

[35] Badeer H.S. (2001). Hemodynamics for medical students. Physiol. Educ..

[36] Howell G.W., Weathers T.M. (1970). Aerospace Fluid Component Designers’ Handbook.

[37] Mott R.L., Untener J.A. (2018). Applied Fluid Mechanics.

