# Supplementary material for: Fractional Flow Reserve Measurement Using Dynamic CT Perfusion Imaging in Patients with Heavy Coronary Calcification and Stent
Source: Diagnostics (Basel). 2026 Jul 31;16(15):2408. doi: 10.3390/diagnostics16152408 (PMC13463942; doi:10.3390/diagnostics16152408)
Supplement: Supplementary file 1 [file diagnostics-16-02408-s001.zip › diagnostics-4260558-supplementary.pdf]

Supplementary Materials

A detailed description of the CT-DAI analytic algorithm is provided in this section.

Step 1: Calculate the flow velocities across the stenosis

The indicator-dilution principle[34] is employed to estimate the volumetric flow rate in the artery of interest. The mathematical formulation of the indicator-dilution principle is as follows:

$$F = \frac{m}{\int_0^T C_a(t)dt} \tag{S1}$$

Where F represents the volumetric flow rate in the artery, m denotes the mass of iodine present in the artery, and $C_a(t)$ signifies the iodine concentration at time t. The integral of $C_a(t)$ from time zero (before contrast arrival) to time T (the end of first-pass circulation) can be approximated from a coronary time-enhancement curve sampled via dynamic myocardial CT perfusion imaging, incorporating appropriate unit conversion from Hounsfield Units (HU) to iodine concentration corresponding to the X-ray energy used for imaging. The volumetric flow rate at any point within the artery is subsequently converted into flow velocity through the following equation:

$$V = \frac{F}{\pi r^2} \tag{S2}$$

Where V is the flow velocity and r is the lumen radius at the specified point in the artery. To measure the pressure gradient across a stenosis, the flow velocity proximal and distal to a stenosis must be known. In other words, a pair of prelesion and postlesion time-enhancement curves must be acquired.

Step 2: Compute the pressure difference across the stenosis

The pressure gradient between any two selected points, A and B, in a coronary artery can be estimated using Bernoulli's principle, which is grounded in the law of conservation of energy[35]:

$$P_A + \frac{1}{2}\rho {V_A}^2 + \rho g h_A = P_B + \frac{1}{2}\rho {V_B}^2 + \rho g h_B + P_L$$
$$\Delta P = P_B - P_A = \frac{1}{2}\rho\left({V_A}^2 - {V_B}^2\right) + \rho g(h_A - h_B) - P_L \tag{S3}$$

Where P_A and P_B denote the pressures at points A and B, respectively; ρ is the density of blood; g represents the acceleration due to gravity; h_A and h_B indicate the vertical heights relative to a reference point; and V_A and V_B denote the flow velocities at points A and B, respectively. The pressure loss, P_L, between two selected points in an artery can be attributed to the frictional force exerted by blood against the arterial wall and the abrupt changes in lumen cross-sectional area due to the presence of stenosis:

$$P_L \approx P_{LF} + P_{LS} \tag{S4}$$

Where P_{LF} represents the pressure loss due to friction against the arterial wall caused by the movement of blood, and P_{LS} denotes the pressure loss due to a stenosis. P_{LF} can be estimated by employing the Darcy-Weisbach equation:[36]

$$P_{LF} = f\left(\frac{L}{D}\right)\left(\frac{\rho \bar{V}^2}{2}\right) \tag{S5}$$

Where f is the Darcy friction factor; L, D and V represent the distance, the mean lumen diameter and the mean flow velocity between the two points, respectively. The pressure loss arising from a stenosis, P_{LS}, can be further attributed to abrupt lumen constriction (P_{LC}) and expansion (P_{LE}):

$$P_{LS} = P_{LC} + P_{LE} \tag{S6}$$

P_{LC} can be approximated using the following equation:[34]

$$P_{LC} \approx \rho \cdot g \cdot \left[k \cdot \frac{{V_{in}}^2}{2g} \right]$$

(S7)

Where V_{in} is the flow velocity in the narrowed arterial segment; k is the head loss coefficient associated with abrupt lumen constriction. The square bracket term on the right side of Equation 7 signifies the head loss. The head loss coefficient value ranges from 0 to 1, and it was assumed to be 0.5 in our study. With this assumption, Equation 7 can be further simplified as:

$$P_{LC} = \rho \left[\frac{{V_{in}}^2}{4} \right]$$

(S8)

P_{LE} can be approximated using the following equation:[37]

$$P_{LE} = \rho \cdot g \cdot \left[\frac{(V_{in} - V_{exit})^2}{2g} \right]$$
$$= \rho \left[\frac{(V_{in} - V_{exit})^2}{2} \right]$$

(S9)

Where V_{exit} represents the flow velocity immediately after the exit side of a stenosis. It is clear that V_{in} is needed to compute P_{LE} and P_{LC}. However, sampling the time-enhancement curve in a stenosed coronary segment can be challenging, and therefore, V_{in} may not be readily estimated using Equations (1) and (2). Alternatively, V_{in} can be approximated using the flow velocity adjacent to the entry side of a stenosis, V_{entry}, based on the continuity principle:

$$V_{in} \cdot \pi {r_{in}}^2 = V_{entry} \cdot \pi {r_{entry}}^2$$
$$V_{in} = V_{entry} \cdot \left(\frac{r_{entry}}{r_{in}} \right)^2$$

(S10)

Where r_{entry} represents the lumen radius immediately before the entrance of a stenosis and r_{in} denotes the lumen radius of the narrowed coronary artery segment. Time-enhancement curves can be sampled adjacent to the stenosis entry and exit sides to properly estimate the pressure loss associated with the stenosis, P_{LS} , using equations 6 to 10. As explained in Methods, the number of entry/exit time-enhancement curves required for FFR measurement depends on the number of stenoses between the selected prelesion and postlesion sites in the coronary artery.

Step 3: Compute the fractional flow reserve

The pressure in the proximal segment of a coronary artery (P_A) is assumed to be identical to the aortic pressure. In this study, systolic blood pressure (SBP) was used as P_A because the dynamic myocardial CT perfusion images were acquired at end-systolic phases and the time-enhancement curves sampled from these images were more influenced by the hemodynamics at systole than diastole. FFR was estimated using the following equation:

$$FFR = \frac{P_B}{P_A} = \frac{(P_B - P_A) + P_A}{P_A} = \frac{\Delta P + SBP}{SBP}$$

(S11)

References

34. Klocke FJ, Greene DG, Koberstein RC. Indicator-dilution measurement of cardiac output with dissolved hydrogen. Circ. Res. 1968, 22, 841–853.

35. Badeer HS. Hemodynamics for medical students. Physiol Educ. 2001 Dec;25(1-4):44-52.

36. Howell GW, Weathers TM. Aerospace fluid component designers' handbook. 1970. TRW Systems Group, Redondo beach, Calif. NTIS issue number: 197209.

37. Mott RL, Untener JA. Applied Fluid Mechanics. 7th Edition. 2018. Pearson, London, United Kingdom. ISBN-13: 9781292019611.
